# Internet-Based Cognitive Behavioral Therapy for Preventing Postpartum Depressive Symptoms Among Pregnant Individuals With Depression: Multicenter Randomized Controlled Trial in China

**DOI:** 10.2196/67386

**Published:** 2025-03-04

**Authors:** Chen-Chi Duan, Chen Zhang, Hua-Lin Xu, Jing Tao, Jia-Le Yu, Dan Zhang, Shan Wu, Xiu Zeng, Wan-Ting Zeng, Zhi-Yin Zhang, Cindy-Lee Dennis, Han Liu, Jia-Ying Wu, Ben Willem J Mol, He-Feng Huang, Yan-Ting Wu

**Affiliations:** 1 Obstetrics and Gynecology Hospital, Institute of Reproduction and Development, Fudan University Shanghai China; 2 Research Units of Embryo Original Diseases, Chinese Academy of Medical Sciences (No. 2019RU056) Shanghai China; 3 Shaoxing Maternity and Child Health Care Hospital Shaoxing China; 4 Shanghai Mental Health Center Shanghai Jiao Tong University School of Medicine Shanghai China; 5 International Peace Maternity and Child Health Hospital, Shanghai Jiao Tong University School of Medicine Shanghai China; 6 Key Laboratory of Reproductive Genetics (Ministry of Education), Department of Reproductive Endocrinology, Women's Hospital, Zhejiang University School of Medicine Hangzhou China; 7 Department of Gynecology, The Second Affiliated Hospital of Zhejiang University School of Medicine Hangzhou China; 8 Hunan Maternal and Child Health Care Hospital Changsha China; 9 Hangzhou Women’s Hospital Hangzhou China; 10 Lawrence S. Bloomberg Faculty of Nursing, University of Toronto Toronto, ON Canada; 11 Department of Obstetrics and Gynecology, Monash University Clayton Australia

**Keywords:** antenatal depression, postpartum depression, internet-based cognitive behavioral therapy, randomized controlled trial

## Abstract

**Background:**

Women are particularly vulnerable to depression during pregnancy, which is one of the strongest risk factors for developing postpartum depression (PPD). Addressing antenatal depressive symptoms in these women is crucial for preventing PPD. However, little is known about the effectiveness of internet-based cognitive behavioral therapy (ICBT) in preventing PPD in this high-risk group.

**Objective:**

This study aims to evaluate the short- and long-term effects of ICBT in preventing PPD among women with antenatal depressive symptoms.

**Methods:**

Participants were screened for antenatal depressive symptoms using the Edinburgh Postnatal Depression Scale (EPDS) and randomly allocated (1:1) to either the ICBT group (receiving weekly online modules starting antenatally and continuing into early postpartum) or the control group (observed without treatment). Follow-up assessments were conducted up to 12 months postpartum, and data were analyzed using generalized estimating equations. The primary outcome was the prevalence of depressive symptoms at 6 weeks postpartum. A subgroup analysis based on the severity of antenatal depressive symptoms was also performed. The secondary outcomes included the long-term effects of ICBT on maternal depression, as well as its impact on anxiety, sleep quality, social support, parenting stress, co-parenting relationships, and infant development.

**Results:**

Between August 2020 and September 2021, 300 pregnant individuals were recruited from 5 centers across China. No significant differences were observed in depressive symptoms at 6 weeks postpartum (*P*=.18) or at any longer-term follow-up time points (*P*=.18). However, a post hoc subgroup analysis showed that participants with antenatal EPDS scores of 10-12 in the ICBT group had a lower risk of developing depression during the first year postpartum (odds ratio 0.534, 95% CI 0.313-0.912; *P*=.02), but this was not observed for participants with more severe depression. Additionally, this subgroup demonstrated higher levels of co-parenting relationships (*P*=.02).

**Conclusions:**

Among individuals with antenatal depression, ICBT did not prevent the development of PPD. However, ICBT may be a preferable option for those with mild to moderate antenatal depressive symptoms. Future research is needed to explore modifications to ICBT to address more severe depressive symptoms.

**Trial Registration:**

Chinese Clinical Trial Registry ChiCTR2000033433; https://www.chictr.org.cn/showproj.html?proj=54482

**International Registered Report Identifier (IRRID):**

RR2-10.1186/s13063-022-06728-5

## Introduction

Postpartum depression (PPD), which includes major and minor depressive episodes occurring within 1 year postpartum, is a common maternal complication following childbirth, with clear evidence demonstrating both short- and long-term negative effects on mothers, children, family members, and society [1-3]. It affects approximately 20% of pregnant individuals in low- and middle-income countries [4]. Notably, depression during pregnancy is one of the strongest risk factors for PPD, with approximately one-third of individuals with PPD reporting that their symptoms began antenatally [5]. Furthermore, pregnant individuals have been reported to be particularly vulnerable to depression and anxiety since the COVID-19 pandemic, with an increased likelihood of experiencing thoughts of self-harm [6-8]. This heightened prevalence places a significant burden on the existing health care system and the provision of evidence-based treatment [9]. However, the rates of depression diagnosis and treatment uptake among pregnant individuals are lower than those observed in the general population [10,11]. It is also well-documented that pregnant individuals with depressive symptoms are often reluctant to seek treatment due to individual, community, and health system barriers, particularly in low- and middle-income countries [12,13]. Therefore, addressing antenatal depressive symptoms is critically important for preventing PPD.

Cognitive behavioral therapy (CBT) is a practical and effective treatment for depression in the general population, as it helps individuals change negative thoughts, attitudes, and beliefs while promoting positive emotional and behavioral responses [14]. Evidence suggests that CBT can be initiated during pregnancy to prevent the development of PPD. Compared with traditional face-to-face delivery, internet-based CBT (ICBT) is more accessible and cost-effective, addressing common barriers to help-seeking such as shame, lack of time, transportation challenges, and limited medical resources. In this context, delivering CBT via the internet could be a promising intervention for preventing PPD, particularly after the onset of the COVID-19 pandemic, when social isolation, stigmatization, discrimination, and financial losses were frequently reported.

Although individuals with antenatal depressive symptoms are more likely to develop PPD, most studies evaluating the efficacy of prenatal ICBT for preventing PPD have been designed to recruit general pregnant individuals rather than high-risk groups [15-18]. To date, only 4 single-center randomized controlled trials with relatively small sample sizes have been published on ICBT interventions targeting individuals at high risk of antenatal depression [19-22]. Furthermore, in these studies, participants were screened for depressive symptoms before the third trimester of pregnancy, and the follow-up period was limited to 6 weeks postpartum. However, as pregnancy progresses and the physical burden increases, individuals are more likely to experience frequent urination, joint or pelvic pain, shortness of breath, swelling, constipation, indigestion, and pregnancy-related complications in late pregnancy. The prevalence of depressive symptoms during pregnancy has also been reported to rise over time, increasing from 7.4% in early pregnancy to approximately 12% in mid-to-late pregnancy [23]. Individuals with new-onset depressive symptoms emerging in late pregnancy should receive careful attention. To address these research gaps, we conducted this multicenter trial among individuals with antenatal depressive symptoms in their third trimester to evaluate the effect of ICBT on preventing depression across the first year postpartum.

## Methods

### Study Design and Setting

We conducted a 2-arm, parallel-group randomized controlled trial across 5 medical centers in 4 different cities in China, adhering to the CONSORT (Consolidated Standards of Reporting Trials) guidelines (Multimedia Appendix 1 [24]). The trial was carried out through obstetric clinics at the International Peace Maternity and Child Health Hospital in Shanghai, along with 4 collaborating medical centers: Shaoxing Maternity and Child Health Care Hospital in Shaoxing, Hunan Maternal and Child Health Care Hospital in Changsha, the Women’s Hospital affiliated with Zhejiang University School of Medicine in Hangzhou, and Hangzhou Women’s Hospital in Hangzhou.

### Ethical Considerations

Ethics approval was obtained from the institutional review board of the International Peace Maternity and Child Health Hospital (approval number GKLW2020-25). Informed consent was obtained from all participants, and those who completed the trial were financially compensated. To protect participants’ privacy, each individual’s name was replaced with a computer-generated serial number for identification. The trial was registered with the Chinese Clinical Trial Registry (ChiCTR2000033433), and the detailed protocol has been published [25].

### Participant Screening and Recruitment

We screened pregnant individuals for antenatal depressive symptoms using the Edinburgh Postnatal Depression Scale (EPDS) during their third trimester of pregnancy (≥28 and ≤34 gestational weeks) as part of a standardized visit, which all pregnant individuals receive at the 5 participating medical centers. The EPDS is the most widely used screening tool for perinatal depression globally, demonstrating good sensitivity and specificity among Chinese individuals when a cut-off score of ≥10 is applied, as recommended for Asian populations [26]. Additionally, a higher cut-off point of ≥13 on the EPDS, indicating a higher level of depressive symptoms, is recommended in settings with limited resources for further treatment [27,28]. Pregnant individuals were eligible to participate in the trial if they met the following inclusion criteria: having a singleton fetus, being 18 years of age or older, being between ≥28 and ≤37 gestational weeks, being able to read and understand Chinese, having an EPDS score ≥10, and having access to the internet via a smartphone or computer. Individuals were excluded if they had active suicidal ideation, a diagnosis of severe mental illness (eg, schizophrenia), or were currently receiving treatment for depression. Eligible individuals were then contacted and introduced to the study, and those who agreed to participate were asked to complete the informed consent procedures and the baseline questionnaire with the assistance of a research assistant.

### Randomization, Blinding, and Enrollment

All eligible individuals were ensured enrollment before 37 gestational weeks by a trained research assistant who was not involved in any other trial activities. Participants were randomly allocated in a 1:1 ratio to either the intervention group or the control group using a computer-generated block randomization procedure provided by the Clinical Research Public Technical Service Platform [29]. The allocation sequence was implemented through a web-based central randomization system, and the block size for randomization was set to 6. Based on the results provided by the computer-generated randomization system, participants were asked to register in the perinatal health care app. They were then assigned to either the intervention or control group by the research assistant. Because of the nature of the intervention, both the research assistant and the therapist providing the ICBT intervention were not blinded to group allocation; however, participants were blinded. To maintain blinding, all eligible individuals were thoroughly informed about the online courses and follow-up assessments, but were not told about the differences in courses provided between the 2 groups.

### Treatment

#### Control Group

**Figure 1:**
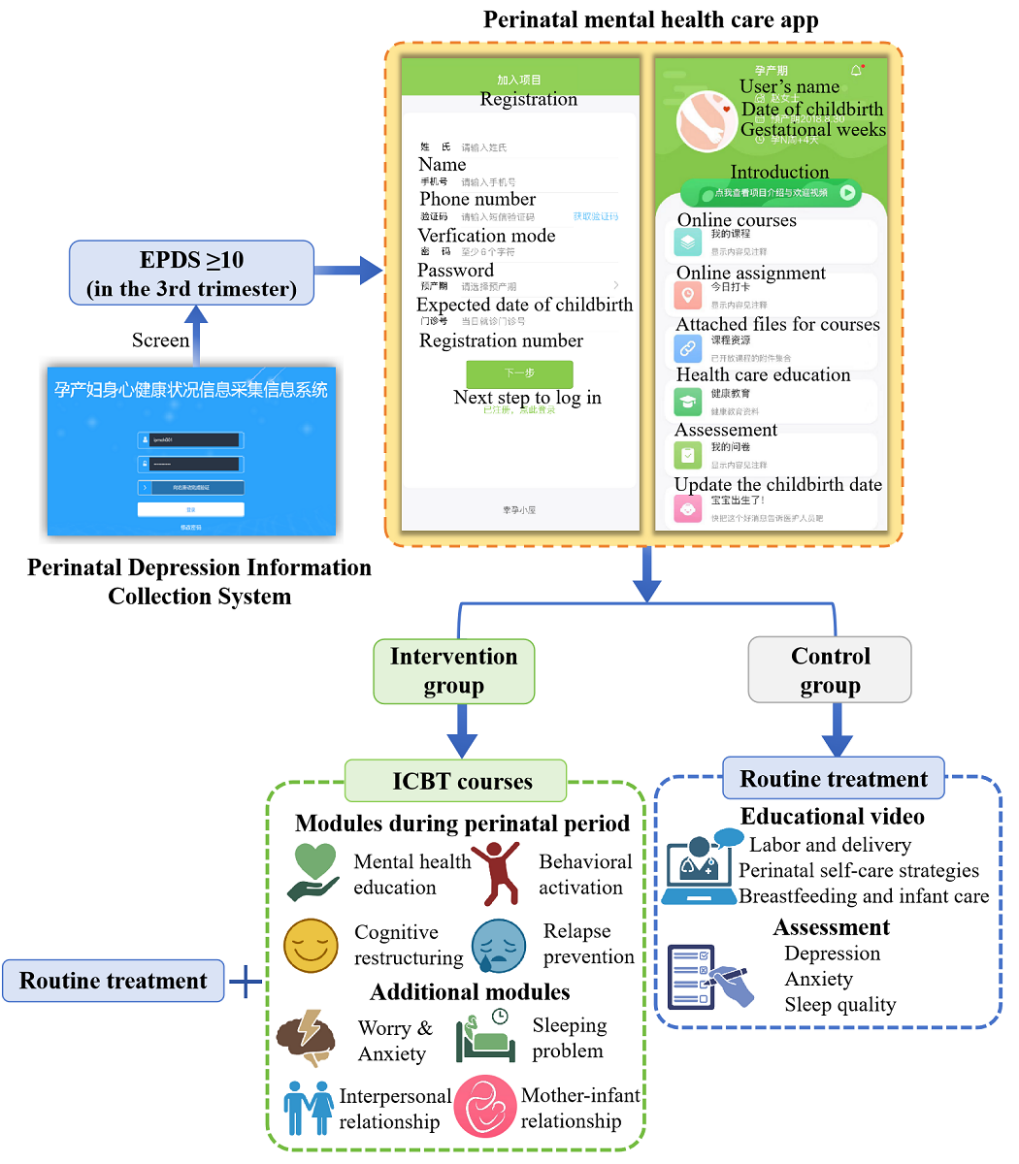
Flow diagram of participant recruitment and treatment for both groups. ICBT: internet-based cognitive behavioral therapy; EPDS: Edinburgh Postnatal Depression Scale.

Participants in the control group received standard antenatal care, including educational videos and timely depression assessments delivered through the perinatal health care app developed for the trial (Figure 1). The videos provided information on labor and delivery, postpartum self-care, infant care, and breastfeeding. The content of these videos, which is also commonly provided in prenatal courses at medical centers, was not related to depression. Additionally, via the app, participants in the control group were assessed for depressive symptoms using the 9-item Patient Health Questionnaire (PHQ-9) once every 2 weeks before childbirth. It has been reported that the detection rate of prenatal depression measured by the EPDS is relatively higher compared with the PHQ-9 [30], suggesting that the EPDS may have greater sensitivity in screening for depressive symptoms. However, the PHQ-9 has an advantage over the EPDS in distinguishing the severity of depressive symptoms, with scores of ≥5, ≥10, and ≥15 corresponding to mild, moderate, and severe levels of depression, respectively [31]. Individuals with a total PHQ-9 score of ≥15 or a score of ≥2 on the self-harm item were referred to a registered psychiatrist for further assessment.

#### Intervention Group

Participants in the intervention group received standard antenatal care as described above, along with online text and video-based modules provided weekly through the perinatal health care app (Figure 1); 3 modules were delivered during pregnancy, and 4 modules were delivered from 2 to 5 weeks postpartum. Participants were required to complete assignments and provide feedback to the therapist via the app after each module was published. If participants had not viewed the weekly modules or submitted feedback, they would receive an automatic reminder message through the app, as well as a personal contact from the therapist. Therapists were graduate-level trainees in psychology, supervised by a registered psychiatrist with experience in treating depression. The training included a 2-day workshop before they began the intervention, and therapists were also provided with a manual outlining the weekly content for ICBT. In addition to regular weekly courses on psychoeducation, cognitive restructuring, problem-solving strategies, behavioral activation, and relapse prevention, 4 additional modules were provided. These modules addressed maternal anxiety and worries, sleep problems, interpersonal relationship difficulties, and mother-infant relationship development.

### Outcomes

#### Overview

All study participants completed follow-up questionnaires via the app at 6 weeks postpartum, followed by additional questionnaires at 3, 6, 9, and 12 months postpartum.

#### Primary Outcome

The primary outcome was the prevalence of depressive symptoms at 6 weeks postpartum, measured using the PHQ-9, a 9-item self-report scale with well-documented psychometric properties and a cut-off score of ≥5 indicating possible depression [31].

#### Secondary Outcomes

To further evaluate the longer-term effect of ICBT treatment on preventing the development of depressive symptoms, the PHQ-9 was readministered at 3, 6, and 12 months postpartum.

Anxiety was assessed using the 7-item Generalized Anxiety Disorder (GAD-7), a scale measuring symptoms over the past 2 weeks. Total scores range from 0 to 21, with a cut-off score >4 suggesting possible anxiety, and higher scores indicating more severe symptoms [32]. The GAD-7 was administered at 6 weeks, and at 3, 6, and 12 months postpartum.

Sleep quality was assessed using the Pittsburgh Sleep Quality Index (PSQI), a 19-item scale with good psychometric properties, measuring sleep quality over the past month [33]. The PSQI evaluates 7 key aspects of sleep, including subjective sleep quality, sleep latency, sleep duration, habitual sleep efficiency, sleep disturbances, use of sleep medication, and daytime dysfunction. Total scores range from 0 to 21, with higher scores indicating worse sleep quality. A cut-off score >5 is recommended to distinguish between good and poor sleep quality [33]. The PSQI was administered at 3, 6, and 12 months postpartum.

Social support was assessed using the Multidimensional Scale of Perceived Social Support (MSPSS), a 12-item scale that measures the perceived availability and adequacy of emotional and instrumental social support. Total scores range from 12 to 84, with higher scores indicating greater levels of perceived support [34]. The MSPSS was administered at 6 months postpartum.

Parenting stress was assessed using the Parenting Stress Index-Short Form (PSI-SF), a 36-item scale that measures parenting stress. It consists of 3 subscales: Parental Distress, Parent-Child Dysfunctional Interaction, and Difficult Child, with scores ranging from 12 to 60. Total scores are calculated by summing the 3 subscale scores, with scores of ≥90 indicating high levels of parenting stress [35]. The PSI-SF was administered at 9 months postpartum.

The co-parenting relationship was assessed using the Brief Co-Parenting Relationship Scale (BCRS), a 14-item scale that measures co-parenting dynamics across 7 subscales: co-parenting agreement, co-parenting closeness, exposure to conflict, co-parenting support, co-parenting undermining, endorsement of partner parenting, and division of labor. Lower scores in the subscales of exposure to conflict and co-parenting undermining, and higher scores in the other 5 subscales, indicate a more positive co-parenting relationship [36]. The BCRS was administered at 9 months postpartum.

Infant development was assessed using the Denver Development Screening Test (DDST) II, a developmental assessment tool for infants and children aged 0-6 years. The scale includes items that evaluate gross motor development, fine motor-adaptive development, language development, and personal-social development. Items on the scale that over 90% of children of the same age are expected to pass but are not successfully completed are considered as “developmental delay.” Items that 75%-90% of children of the same age are expected to accomplish but are not completed are scored as a “warning.” Results are categorized as normal (no “developmental delay” or 1 “warning”), abnormal (2 or more “developmental delays”), or suspicious (2 or more “warnings” and 1 “developmental delay”) [37]. The DDST II was administered at 3, 6, and 12 months postpartum.

### Sample Size

The sample size for this study was determined based on a previous Chinese trial, which reported lower rates of PPD following face-to-face CBT treatment among individuals with antenatal depressive symptoms (37/167, 22.2% vs 79/167, 47.3%; *P*=.001) [38]. We calculated a required sample size of at least 178 participants (89 per group, accounting for a 20% loss to follow-up) to detect a 25% reduction in PPD with 90% power and a 2-sided significance level of .05. Considering the potential reduction in efficacy with online CBT compared with face-to-face delivery, we aimed to recruit 300 participants for this study.

### Statistical Analysis

Baseline characteristics were summarized using descriptive statistics. Continuous variables with a normal distribution were reported as means and SDs, while those with a skewed distribution were expressed as medians and IQRs. Categorical variables were presented as frequencies with proportions. Primary and secondary outcomes were analyzed according to the intention-to-treat principle. Group differences in continuous variables with a normal distribution were assessed using the Student *t* test, whereas the Kolmogorov-Smirnov test was applied for variables with a skewed distribution. Group differences in categorical variables at each time point were analyzed using a chi-square test. To evaluate the effect of the ICBT intervention over time, generalized estimating equation modeling with an unstructured correlation structure was used to analyze outcomes measured repeatedly at multiple time points [39]. The generalized estimating equation model was adjusted for covariates selected based on the quasi-likelihood under the independence model criterion. A post hoc subgroup analysis was conducted to further assess the intervention’s effect on preventing PPD among participants with varying severity of depressive symptoms at baseline. All statistical analyses were performed using R software (version 4.0.2; R Foundation), with significance defined as a 2-sided *P* value less than .05.

## Results

**Figure 2:**
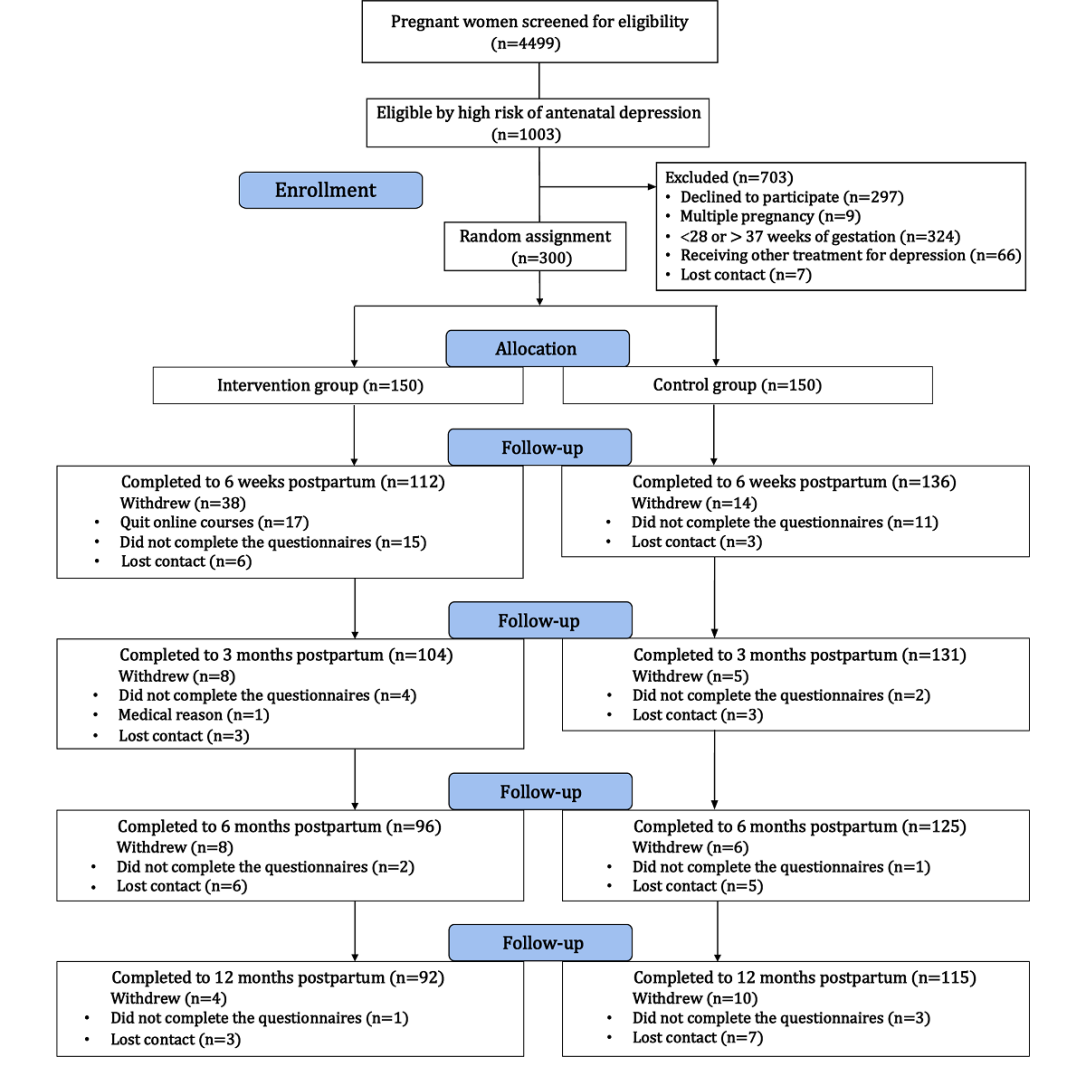
Flowchart of the study participants.

Between August 2020 and September 2021, 4499 pregnant individuals were screened for depressive symptoms, of whom 1003 were identified with an EPDS score ≥10 (Figure 2 and Multimedia Appendix 1 [24]). Among these, 297 declined to participate, 9 were excluded due to multiple pregnancies, 324 were excluded for not being between 28 and 37 weeks of gestation, 66 were excluded for receiving other treatment for depression, and 7 could not be contacted. Ultimately, 300 individuals met the eligibility criteria and provided informed consent. Of these, 150 were randomized to the intervention group and 150 to the control group.

The baseline characteristics are summarized in Table 1. The median maternal age was 29 (IQR 27-32) years, with the majority of participants being college educated, employed, and primiparous. The median antenatal EPDS score was 12 (IQR 11-15), with 157 of 300 (52.3%) participants reporting mild to moderate depressive symptoms, indicated by an EPDS score ≥10 and <13. Participants in the intervention group had a slightly higher median antenatal EPDS score compared with those in the control group. Among the 248 participants included in the final intention-to-treat analysis, no significant differences were observed between the 2 groups regarding baseline depressive symptom severity, maternal sociodemographic characteristics, or pregnancy complications.

**Table 1:** Baseline characteristics of participants from the intervention and control groups.

Characteristics				All participants enrolled in the study							Participants included in the ITT^a^ analysis											
				ICBT^b^ group (n=150)		Control group (n=150)		*P* value		ICBT group (n=112)			Control group (n=136)					*P* value				
**EPDS^c^ assessment at baseline**																						
	Median (IQR)		12 (11.00-15.00)		12 (10.00-14.25)		.03		12 (11.00-14.75)			12 (10.00-14.75)					.15					
	EPDS ≥10 and <13, n (%)		76 (50.67)		81 (54.00)		.56		57 (50.89)			75 (55.15)					.50					
	EPDS ≥13, n (%)		74 (49.33)		69 (46.00)				55 (49.11)			61 (44.85)										
**Maternal sociodemographic characteristics**																						
	Age (years), median (IQR)		30.00 (27.00-32.00)		29.00 (27.00-33.00)		.89		30.00 (27.00-32.00)			29.00 (27.00-32.00)					.92					
	Pregestational BMI (kg/m^2^), median (IQR)		20.31 (18.82-22.29)		20.83 (19.31-22.86)		.18		20.45 (19.03-22.81)			21.09 (19.50-22.89)					.50					
**Location, n (%)**								.98										.81				
	Shanghai		40 (26.67)		41 (27.33)				32 (28.57)			39 (28.68)										
	Hangzhou		52 (34.67)		49 (32.67)				41 (36.61)			43 (31.62)										
	Changsha		34 (22.67)		36 (24.00)				23 (20.54)			30 (22.06)										
	Shaoxing		24 (16.00)		24 (16.00)				16 (14.29)			24 (17.65)										
**Ethnicity, n (%)**								.28										.83				
	Han		144 (96.00)		148 (98.67)				109 (97.32)			134 (98.53)										
	Minority		6 (4.00)		2 (1.33)				3 (2.68)			2 (1.47)										
**Educational attainment, n (%)**								.62										.46				
	High school or lower		29 (19.33)		25 (16.67)				21 (18.75)			19 (13.97)										
	College		104 (69.33)		103 (68.67)				78 (69.64)			96 (70.59)										
	Master or above		17 (11.33)		22 (14.67)				13 (11.61)			21 (15.44)										
**Occupation, n (%)**								.23										.18				
	Employed		87 (58.00)		101 (67.33)				66 (58.93)			95 (69.85)										
	Self-employed		29 (19.33)		21 (14.00)				19 (16.96)			15 (11.03)										
	Unemployed		34 (22.67)		28 (18.67)				27 (24.11)			26 (19.12)										
**Annual household income, n (%)**								.24										.30				
	<US $4000		4 (2.67)		3 (2.00)				3 (2.68)			3 (2.21)										
	US $4001-US $10,000		16 (10.67)		15 (10.00)				13 (11.61)			11 (8.09)										
	US $10,001-US $20,000		43 (28.67)		45 (30.00)				33 (29.46)			40 (29.41)										
	US $20,001-US $40,000		43 (28.67)		58 (38.67)				32 (28.57)			55 (40.44)										
	>US $40,000		44 (29.33)		29 (19.33)				31 (27.68)			27 (19.85)										
Alcohol drinking before pregnancy, n (%)				88 (58.67)		85 (56.67)		.73		63 (56.25)			76 (55.88)					.95				
Alcohol drinking during pregnancy, n (%)				3 (2.00)		2 (1.33)		>.99		2 (1.79)			1 (0.74)					.45				
Smoking before pregnancy, n (%)				16 (10.67)		12 (8.00)		.43		10 (8.93)			10 (7.35)					.65				
Smoking during pregnancy, n (%)				2 (1.33)		1 (0.67)		>.99		1 (0.89)			1 (0.74)					.89				
**History of reproduction**																						
	**Parity, n (%)**						>.99										.92					
		0	104 (69.33)		104 (69.33)				79 (70.54)			99 (72.79)										
		1	44 (29.33)		44 (29.33)				32 (28.57)			36 (26.47)										
		≥2	2 (1.33)		2 (1.33)				1 (0.89)			1 (0.74)										
	**Number of previous abortions, n (%)**						.18										.40					
		0	82 (54.67)		92 (61.33)				66 (58.93)					89 (65.44)								
		1	49 (32.67)		48 (32.00)				36 (32.14)			40 (29.41)										
		≥2	19 (12.67)		10 (6.67)				10 (8.93)			7 (5.15)										
	Previous ectopic pregnancy, n (%)		5 (3.33)		3 (2.00)		.72		3 (2.68)			3 (2.21)					>.99					
	Planned pregnancy, n (%)		85 (56.67)		87 (58.00)		.81		48 (42.86)			54 (39.71)					.62					
	**Pregnancy complications, n (%)**																					
		Gestational diabetes mellitus	24 (16.00)		22 (14.67)		.75		20 (17.86)			23 (16.91)					.72					
		Gestational hypertension	4 (2.67)		4 (2.67)		>.99		3 (2.68)			4 (2.94)					>.99					
		Intrahepatic cholestasis of pregnancy	2 (1.33)		1 (0.67)		.86		2 (1.79)			1 (0.74)					.86					
		Placenta previa	15 (10.00)		8 (5.33)		.13		12 (10.71)			7 (5.15)					.10					
		Fetal growth restriction	3 (2.00)		4 (2.67)		>.99		3 (2.68)			4 (2.94)					>.99					

^a^ITT: intention-to-treat.

^b^ICBT: internet-based cognitive behavioral therapy.

^c^EPDS: Edinburgh Postnatal Depression Scale.

Of the 300 participants enrolled, 52 (17.3%) were lost to follow-up at 6 weeks postpartum, 65 (21.7%) at 3 months postpartum, 79 (26.3%) at 6 months postpartum, and 93 (31.0%) at 12 months postpartum. On average, participants in the ICBT group completed 2.5 (SD 1.1) modules during pregnancy and 3.1 (SD 1.5) modules postnatally. Of the 150 in the ICBT group, 129 (86%), 123 (82%), 129 (86%), 118 (79%), 118 (79%), 117 (78%), and 117 (78%), respectively, completed modules 1-7. Notably, 108 (72%) of participants completed all intervention modules delivered via the perinatal health care app.

**Figure 3:**
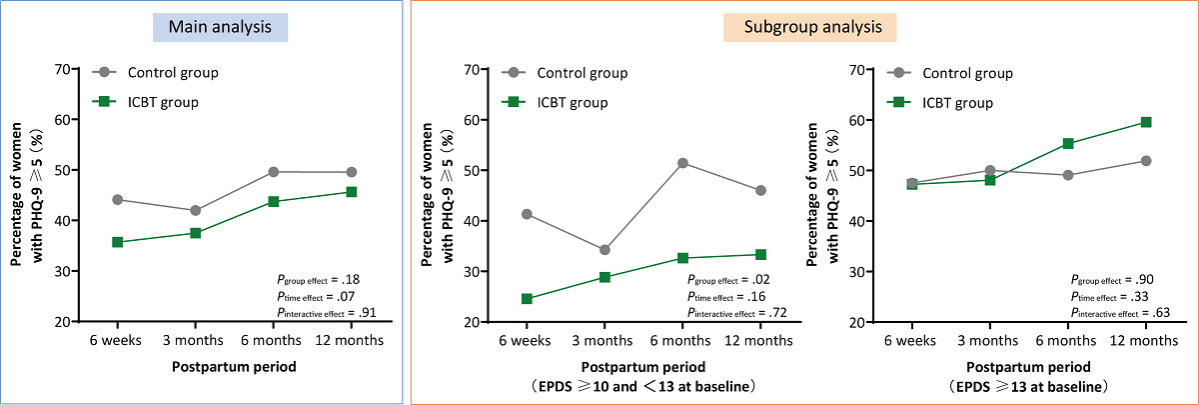
Differences in postpartum depressive symptoms between the control and ICBT groups at each time point. EPDS: Edinburgh Postnatal Depression Scale; ICBT: internet-based cognitive behavioral therapy; PHQ-9: 9-item Patient Health Questionnaire.

The primary outcome, depressive symptoms at 6 weeks postpartum, did not differ significantly between the groups (ICBT vs control group: 40/112, 35.7% vs 60/136, 44.1%; *P*=.18; Table 2). Figure 3 illustrates the trajectory of depressive symptoms from 6 weeks postpartum to 1 year, as measured by the PHQ-9. No significant differences in symptom severity were observed between the intervention and control groups at any follow-up time point (*P*=.18; Figure 3, Table 2). Although symptoms worsened over time, this change was not statistically significant (*P*=.07).

**Table 2:** Differences in outcomes between participants from the ICBT^a^ group and the control group at each time point.

Depressive symptoms							ICBT group				Control group				*P* value
**Primary outcomes**															
	**Depressive symptoms at 6 weeks postpartum**														
		PHQ-9^b^, median (IQR)			3.00 (1.00-7.00)				4.00 (1.00-8.00)				.64		
		PHQ-9≥5, n/N (%)			40/112 (35.71)				60/136 (44.12)				.18		
**Secondary outcomes**															
	**Depressive symptoms**														
		**3 months postpartum**													
			PHQ-9, median (IQR)	3.00 (1.00-7.00)				3.00 (1.00-8.00)				.97			
			PHQ-9≥5, n/N (%)	39/104 (37.50)				55/131 (41.98)				.49			
		**6 months postpartum**													
			PHQ-9, median (IQR)	4.00 (1.00-8.00)				4.00 (0.50-8.00)				.99			
			PHQ-9≥5, n/N (%)	42/96 (43.75)				62/125 (49.60)				.39			
		**3 months postpartum**													
			PHQ-9, median (IQR)	4.00 (1.00-8.00)				3.00 (0.00-9.00)				.37			
			PHQ-9≥5, n/N (%)	42/92 (45.65)				57/115 (49.57)				.57			
	**Anxiety symptoms**														
		**6 weeks postpartum**													
			GAD-7^c^, median (IQR)	3.00 (0.00-6.00)				3.00 (0.00-7.00)				.98			
			GAD-7≥5, n/N (%)	40/112 (35.71)				54/136 (39.71)				.52			
		**3 months postpartum**													
			GAD-7, median (IQR)	3.00 (0.00-6.00)				3.00 (0.00-6.00)				.99			
			GAD-7≥5, n/N (%)	35/104 (33.65)				46/131 (35.11)				.81			
		**6 months postpartum**													
			GAD-7, median (IQR)	3.00 (0.00-6.00)				3.00 (0.00-6.50)				.98			
			GAD-7≥5, n/N (%)	38/96 (39.58)				49/125 (39.20)				.95			
		**12 months postpartum**													
			GAD-7, median (IQR)	3.00 (0.00-6.00)				3.00 (0.00-6.00)				.88			
			GAD-7≥5, n/N (%)	38/92 (41.30)				43/115 (37.39)				.57			
		**Sleep quality**													
			PQSI^d^≥6 (3 months postpartum), n/N (%)	86/104 (82.69)				111/131 (84.73)				.67			
			PQSI≥6 (6 months postpartum), n/N (%)	78/96 (81.25)				96/125 (76.80)				.42			
			PQSI≥6 (12 months postpartum), n/N (%)	67/92 (72.83)				85/115 (73.91)				.86			

^a^ICBT: internet-based cognitive behavioral therapy.

^b^PHQ-9: 9-item Patient Health Questionnaire.

^c^GAD-7: 7-item Generalized Anxiety Disorder.

^d^PSQI: Pittsburgh Sleep Quality Index.

Interestingly, in a subgroup analysis of participants with a baseline EPDS score ≥10 and <13, those in the intervention group had significantly lower levels of postpartum depressive symptoms (odds ratio 0.534, 95% CI 0.313-0.912; *P*=.02; Table 3). The differences in depressive symptoms were observed at 6 weeks postpartum (ICBT vs control group: 14/57, 25% vs 31/75, 41%; *P*=.04) and at 6 months postpartum (ICBT vs control group: 16/49, 33% vs 36/70, 51%; *P*=.04; Figure 3 and Table S2 in Multimedia Appendix 2). After adjusting for covariates, the subgroup analysis indicated that participants in the intervention group were less likely to develop postpartum depressive symptoms compared with those in the control group (adjusted odds ratio 0.577, 95% CI 0.330-0.965; *P*=.04; Table 3).

**Table 3:** Differences in outcomes between participants from the ICBT^a^ group and the control group at each time point using the GEE^b^ model.

ICBT group versus control group					Odds ratio (95% CI)			*P* value			Adjusted odds ratio (95% CI)			*P* value
Depressive symptoms^c^					0.758 (0.505-1.136)			.18			0.735 (0.487-1.112)^d^			.14^d^
Anxiety symptoms^e^					0.948 (0.633-1.420)			.80			0.915 (0.608-1.376)^f^			.67^f^
Sleep quality^g^					1.010 (0.605-1.686)			.97			0.943 (0.558-1.592)^f^			.82^f^
**Subgroup analysis**														
	**EPDS^h^ ≥10 and <13 at baseline**													
		Depressive symptoms^i^	0.534 (0.313-0.912)			.02			0.577 (0.330-0.965)^j^			.04^j^		
		Anxiety symptoms^k^	0.838 (0.466-1.471)			.54			0.928 (0.529-1.629)^l^			.79^l^		
		Sleep quality^m^	1.010 (0.510-2.000)			.98			1.387 (0.692-2.786)^n^			.36^n^		
	**EPDS ≥13 at baseline**													
		Depressive symptoms^i^	0.962 (0.524-1.764)			.90			0.943 (0.511-1.742)^j^			.85^j^		
		Anxiety symptoms^k^	0.969 (0.544-1.724)			.91			0.896 (0.472-1.701)^o^			.74^o^		
		Sleep quality^m^	0.951 (0.442-2.053)			.90			0.674 (0.293-1.550)^p^			.35^p^		

^a^ICBT: internet-based cognitive behavioral therapy.

^b^GEE: generalized estimating equation.

^c^Defined as PHQ-9≥5 measured at 6 weeks, 3 months, 6 months, and 1 year postpartum.

^d^Values were adjusted for occupation and location.

^e^Defined as GAD-7≥5 measured at 6 weeks, 3 months, 6 months, and 1 year postpartum.

^f^Values were adjusted for location.

^g^Defined as PQSI≥6 measured at 3 months, 6 months, and 1 year postpartum.

^h^EPDS: Edinburgh Postnatal Depression Scale.

^i^*P* value for interaction ^i^*P*=.002.

^j^Values were adjusted for occupation and intrahepatic cholestasis of pregnancy.

^k^*P* value for interaction ^k^*P*<.001.

^l^Values were adjusted for smoking before pregnancy and educational attainment.

^m^*P* value for interaction ^i^*P*=.09.

^n^Values were adjusted for location and education.

^o^Values were adjusted for location, occupation, intrahepatic cholestasis of pregnancy, and gestational diabetes mellitus.

^p^Values were adjusted for location, occupation, and educational attainment.

No significant differences between groups were found over time for the secondary outcomes of anxiety and sleep quality (Table 2). Additionally, there were no significant group differences related to social support, the co-parenting relationship, parenting satisfaction, or infant development (see Table S1 in Multimedia Appendix 2). However, subgroup analyses of participants with a baseline EPDS score between ≥10 and <13 revealed that those in the intervention group had significantly higher perceptions of co-parenting compared with the control group (ICBT vs control group: mean 7.86, SD 0.52 vs mean 6.35, SD 0.42; *P*=.02; see Table S3 in Multimedia Appendix 2). Although not statistically significant, subgroup analyses also suggested that participants in the intervention group had a higher proportion of infants classified with normal development than those in the control group at 1 year postpartum (ICBT vs control group: 37/45, 82% vs 46/63, 73%; *P*=.08).

## Discussion

### Principal Findings

This is the largest randomized controlled trial with a multicenter design to evaluate the effect of ICBT in treating depressive symptoms among pregnant participants and preventing the development of PPD. The results suggest that while lower rates of depressive symptoms were observed in the intervention group during the first year postpartum, the findings were not conclusive. However, in a subgroup of pregnant participants with mild to moderate depressive symptoms at baseline, those in the intervention group were significantly less likely to experience depressive symptoms at 6 weeks and 6 months postpartum compared with the control group. Participants in the intervention group were also more likely to report higher levels of co-parenting. These findings suggest that ICBT may be more effective for pregnant participants with mild to moderate depressive symptoms compared with those with more severe symptomatology.

### Comparison With Prior Work

Pregnant individuals with depressive symptoms are at high risk of experiencing a continuation of these symptoms into the postpartum period, often with increasing severity. However, few studies have specifically targeted this vulnerable group with the goal of preventing the development of depressive symptoms during the first year postpartum, and the results have been inconsistent. Both Sun et al [21] (N=168) and Forsell et al [20] (N=42) found ICBT to be effective in alleviating maternal depressive symptoms at 6 weeks postpartum among those with antenatal depressive symptoms. Similar positive results were reported in a pilot study (N=25); however, individuals with major antenatal depressive symptoms were excluded from this study [19]. By contrast, Heller et al [22] (N=159) did not observe a statistically significant reduction in postpartum symptomatology following ICBT treatment among pregnant individuals with moderate to severe depressive symptoms. One potential explanation for these inconsistent findings is the timing of depressive symptom identification. Previous studies identified pregnant individuals with depressive symptoms early in the first and second trimesters (<28 gestational weeks). Our trial was the first to initiate ICBT in the third trimester and continue into the early postpartum period, reinforcing antenatal content and addressing transitional stressors immediately after delivery. However, the timing of treatment implementation may have been too late to effectively reduce symptoms and prevent the development of depressive symptoms later in the postpartum period.

Another possible explanation for the overall nonsignificant intervention effect may be an overestimation of the effectiveness of ICBT in improving severe depressive symptoms. Our findings suggest that the ICBT intervention was beneficial for pregnant individuals with mild to moderate symptoms but not for those with more severe symptomatology (baseline EPDS≥13). Similarly, a recent large Japanese trial (N=5017) investigating the effectiveness of ICBT in preventing the onset of major depressive episodes in the third trimester and at 3 months postpartum found no overall beneficial intervention effect [17]. However, the results suggest that ICBT may be beneficial in preventing perinatal depression among pregnant individuals with nonmajor depressive symptoms. Similarly, a German trial involving 406 adults with nonmajor depression found that the use of web-based CBT, compared with enhanced usual care, reduced the incidence of major depressive symptoms over 12 months [40]. These findings align with our trial results and indicate that the effectiveness of ICBT may vary depending on the severity of depressive symptoms. Other trials have demonstrated the benefits of ICBT in preventing major depression and reducing severe depressive symptoms in nonperinatal populations, often after intentionally excluding participants with severe symptomatology due to the perceived need for more intensive treatment or pharmacotherapy [41]. Further research is needed to identify individuals who might benefit most from ICBT.

No significant intervention effects were observed for any secondary outcomes, except for co-parenting support. Co-parenting support plays a crucial role in establishing an effective co-parenting relationship, which is essential for reducing mothers’ child-rearing burden and fostering bonding with the infant. Previous studies have also suggested that better co-parenting may lead to a reduction in postpartum depressive symptoms [42]. The effect of ICBT on postpartum anxiety symptoms remains mixed [20-22], while sleep quality improved over time in both groups, likely due to changes in infant sleep and breastfeeding patterns. Although child development was an exploratory outcome in our trial and the study was not powered to detect group differences, we observed a trend suggesting that ICBT may positively influence child development at 1 year postpartum. Previous research has demonstrated that outcomes improve among older children when parents with depression receive intensive treatment interventions [43,44]. However, the mechanisms by which these interventions lead to improvements in child outcomes are complex. Given the evidence showing the adverse effects of PPD on child developmental trajectories [45], interventions may improve child development through various mediators, including enhanced displays of affect, strategies for addressing marital and family conflicts, and improvements in parenting behaviors. These factors may also contribute to alleviating maternal depressive symptoms. Further research evaluating technology-delivered treatments with sufficient power to detect group differences is warranted.

It is important to note that the effect of ICBT on preventing PPD should be interpreted conservatively. PPD differs from other common types of depression, as individuals face unique challenges arising from a combination of physiological and psychological changes during pregnancy, after childbirth, and throughout breastfeeding. The mental health issues resulting from these specific circumstances may exceed the expected effects of traditional ICBT treatment. To address these challenges, we included 4 additional online modules offering self-help materials and strategies for coping with maternal anxiety and worries, sleep problems, interpersonal relationship difficulties, and enhancing the mother-infant relationship. Although these modules were able to address the main concerns of perinatal individuals, they were not directly related to depression. It would have been beneficial to interview the participants to further assess the extent to which ICBT or other mediators in the intervention contributed to alleviating postpartum depressive symptoms.

Our study has several strengths. It is the first to evaluate both the short- and long-term effects of ICBT on reducing depressive symptoms and preventing PPD among pregnant individuals identified through systematic screening at the end of the second trimester. Compared with previous studies aimed at preventing PPD among pregnant individuals with depressive symptoms, our sample is larger and was recruited from multiple sites across China, increasing the generalizability of the findings. While ICBT has been shown to be effective in alleviating depressive and anxiety symptoms among the general adult population during the COVID-19 pandemic [46], only 1 [47] study has focused on maternal depression and evaluated the efficacy of ICBT treatment delivered in a single session. Our study builds on this work and provides evidence that ICBT may be an effective treatment for individuals with mild to moderate antenatal depressive symptoms, with longer-term benefits observed up to 6 months postpartum.

### Limitations

One limitation of this study is that the assessment of depressive and anxiety symptoms relied on self-report rather than a structured diagnostic interview. Although the measures used are internationally recommended and widely adopted, they do not provide a formal diagnosis. A second limitation is that the sample size was based on a previous study showing lower rates of PPD among Chinese pregnant individuals with antenatal depressive symptoms following face-to-face CBT treatment [38]. As a result, our sample size may have been insufficient to examine the secondary and longer-term outcomes. Additionally, in the intervention group, slightly higher baseline EPDS scores were observed, and there was a higher dropout rate, as completing the intervention was more time-consuming for participants. This may have introduced bias into the results. Furthermore, it is difficult to determine whether the COVID-19 pandemic had a more specific and far-reaching impact on the development of PPD, which limits the generalizability of the estimated ICBT intervention effect after the pandemic ended.

### Conclusions

Despite these limitations, our results indicate a beneficial effect of ICBT on treating mild to moderate antenatal depressive symptoms and preventing the development of depressive symptoms later in the postpartum period. ICBT may be an affordable alternative for pregnant individuals with mild to moderate depressive symptoms, compared with those with more severe symptomatology (EPDS≥13). Future research is warranted to expand the appropriate application of ICBT for individuals with mild to moderate depressive symptoms and to explore best practices for optimizing engagement and integrating ICBT into care pathways.

## Appendix

Multimedia Appendix 1 CONSORT checklist.

Multimedia Appendix 2 Additional analysis.

## Abbreviations

BCRS: Brief Co-Parenting Relationship Scale
CBT: cognitive behavioral therapy
CONSORT: Consolidated Standards of Reporting Trials
DDST: Denver Development Screening Test
EPDS: Edinburgh Postnatal Depression Scale
GAD: 7-item Generalized Anxiety Disorder
ICBT: internet-based cognitive behavioral therapy
MSPSS: Multidimensional Scale of Perceived Social Support
PHQ-9: 9-item Patient Health Questionnaire
PPD: postpartum depression
PSI-SF: Parenting Stress Index-Short Form
PSQI: Pittsburgh Sleep Quality Index

## Data Availability

Further access to participant-level data sets or statistical codes is available from the corresponding author on reasonable request.
