# Supplementary material for: Internet-Based Cognitive Behavioral Therapy for Preventing Postpartum Depressive Symptoms Among Pregnant Individuals With Depression: Multicenter Randomized Controlled Trial in China
Source: J Med Internet Res. 2025 Mar 4;27:e67386. doi: 10.2196/67386 (PMC11920666; doi:10.2196/67386)
Supplement: Multimedia Appendix 2 [file jmir_v27i1e67386_app2.docx]

| Table S1. Differences in other secondary outcomes between participants from ICBT group and control group | | | |
| --- | --- | --- | --- |
| **Secondary outcomes** | **ICBT group** | **Control group** | **P value** |
| **Social support at 6 months postpartum** |  |  |  |
| MSPSS, Mean ± SD | 60.18±1.31 | 61.41±1.24 | 0.500 |
| **Co-parenting relationship at 9 months postpartum** | | | |
| BCRS, Mean± SD |  |  |  |
| Co-parenting agreement | 7.82±0.31 | 7.98±0.26 | 0.680 |
| Co-parenting closeness | 6.79±0.35 | 6.93±0.29 | 0.771 |
| Co-parenting support | 6.98±0.40 | 6.34±0.33 | 0.215 |
| Endorse partner parenting | 8.40±0.34 | 8.43±0.30 | 0.945 |
| Division of labor | 7.54±0.35 | 7.74±0.27 | 0.646 |
| Exposure to conflict | 2.91±0.27 | 2.47±0.19 | 0.169 |
| Co-parenting undermining | 2.99±0.31 | 2.68±0.24 | 0.415 |
| **Parenting satisfaction** |  |  |  |
| PSI-SF < 90 (9 months postpartum) | 50/87 (57.47) | 77/120 (64.17) | 0.329 |
| **Infant development** | | | |
| DDST, No.**/** Total No. (%) |  |  |  |
| 3 months postpartum |  |  |  |
| Normal | 82/102 (80.39) | 88/128 (68.75) | 0.127 |
| Suspicious | 17/102 (16.67) | 35/128 (27.34) |  |
| Abnormal | 3/102 (2.94) | 5/128 (3.91) |  |
| 6 months postpartum |  |  |  |
| Normal | 54/94 (57.45) | 64/119 (53.78) | 0.858 |
| Suspicious | 36/94 (38.30) | 49/119 (41.18) |  |
| Abnormal | 4/94 (4.25) | 6/119 (5.04) |  |
| 12 months postpartum |  |  |  |
| Normal | 71/90 (78.89) | 80/112 (71.43) | 0.474 |
| Suspicious | 15/90 (16.67) | 25/112 (22.32) |  |
| Abnormal | 4/90 (4.44) | 7/112 (6.25) |  |
| MSPSS, Multidimensional Scale of Perceived Social Support; PSI-SF, Parenting Stress Index-Short Form; BCRS, Brief Co-parenting Relationship Scale; DDST, Denver Development Screen Test; SD, standard deviations;. | | | |

| Table S2. Subgroup analysis of differences in depressive symptoms between participants from two groups at each time point | | | |
| --- | --- | --- | --- |
| **Depressive symptoms** | **ICBT group**  No.**/** Total No. (%) | **Control group**  No.**/** Total No. (%) | **P value** |
| **EPDS ≥10 and <13 at baseline** |  |  |  |
| 6 weeks postpartum |  |  |  |
| PHQ-9 Median (IQR) | 2.00 (0.00, 4.50) | 3.00 (0.00, 7.00) | 0.107 |
| PHQ-9 ≥ 5 | 14/57 (24.56) | 31/75 (41.33) | **0.044** |
| 3 months postpartum |  |  |  |
| PHQ-9 Median (IQR) | 2.00 (0.00, 5.00) | 2.00 (0.50, 6.00) | 0.917 |
| PHQ-9 ≥ 5 | 14/52 (26.92) | 25/73 (34.25) | 0.384 |
| 6 months postpartum |  |  |  |
| PHQ-9 Median (IQR) | 3.00 (1.00, 6.00) | 4.50 (0.00, 7.00) | 0.395 |
| PHQ-9 ≥ 5 | 16/49 (32.65) | 36/70 (51.43) | **0.042** |
| 12 months postpartum |  |  |  |
| PHQ-9 Median (IQR) | 4.00 (0.00, 6.00) | 4.00 (0.00,7.00) | 0.603 |
| PHQ-9 ≥ 5 | 14/45 (31.11) | 30/63 (47.62) | 0.085 |
| **EPDS ≥13 at baseline** |  |  |  |
| 6 weeks postpartum |  |  |  |
| PHQ-9 Median (IQR) | 4.00 (2.00, 9.00) | 4.00 (1.00, 9.00) | 1.000 |
| PHQ-9 ≥ 5 | 26/55 (47.27) | 29/61 (47.54) | 0.977 |
| 3 months postpartum |  |  |  |
| PHQ-9 Median(IQR) | 4.00 (1.00, 8.00) | 5.00 (1.75, 9.25) | 0.991 |
| PHQ-9 ≥ 5 | 25/52 (48.08) | 30/58 (51.72) | 0.702 |
| 6 months postpartum |  |  |  |
| PHQ-9 Median (IQR) | 5.00 (2.00, 10.00) | 4.00 (1.00, 10.00) | 0.696 |
| PHQ-9 ≥ 5 | 26/47 (55.32) | 26/55 (47.27) | 0.418 |
| 12 months postpartum |  |  |  |
| PHQ-9 Median (IQR) | 5.00 (0.00, 7.00) | 5.50 (1.00, 10.00) | 0.737 |
| PHQ-9 ≥ 5 | 28/47 (59.57) | 27/52 (51.92) | 0.444 |
| PHQ-9, Patient Health Questionnaire 9-item scale; EPDS, Edinburgh Postnatal Depression Scale; IQR, Inter-quartile range. | | | |

| Table S3. Subgroup analysis of differences in other secondary outcomes between participants from two groups at each time point | | | | | | |
| --- | --- | --- | --- | --- | --- | --- |
|  | **Subgroup analysis** | | | | | |
|  | **EPDS ≥10 and <13 at baseline** | | | **EPDS ≥13 at baseline** | | |
| **Secondary outcomes** | **ICBT group**  No. (%) | **Control group**  No. (%) | **P value** | **ICBT group**  No. (%) | **Control group**  No. (%) | **P value** |
| **Anxiety symptoms** |  |  |  |  |  |  |
| 6 weeks postpartum |  |  |  |  |  |  |
| GAD-7 Median (IQR) | 2.00 (0.00, 4.00) | 2 (0.00, 6.00) | 0.726 | 4 (1.00, 8.00) | 5 (1.00, 7.00) | 0.755 |
| GAD-7 ≥ 5 | 13/57 (22.81) | 23/75 (30.67) | 0.315 | 27/55 (49.09) | 31/61 (50.82) | 0.852 |
| 3 months postpartum |  |  |  |  |  |  |
| GAD-7 Median (IQR) | 2.00 (0.00, 4.75) | 2 (0.00, 5.00) | 0.998 | 3 (0.00, 7.00) | 4 (1.00, 7.00) | 0.957 |
| GAD-7 ≥ 5 | 13/52 (25.00) | 20/73 (27.40) | 0.764 | 24/52 (46.15) | 27/58 (41.38) | 0.843 |
| 6 months postpartum |  |  |  |  |  |  |
| GAD-7 Median (IQR) | 3.00 (0.00, 5.00) | 2.5 (0.00, 5.25) | 0.798 | 5 (0.00, 9.00) | 5 (0.00, 7.00) | 0.824 |
| GAD-7 ≥ 5 | 14/49 (28.57) | 22/70 (31.43) | 0.738 | 24/47 (51.06) | 27/55 (49.09) | 0.843 |
| 12 months postpartum |  |  |  |  |  |  |
| GAD-7 Median (IQR) | 2.00 (0.00, 5.50) | 2 (0.00, 6.00) | 0.995 | 5 (0.00, 7.00) | 4 (1.00, 7.00) | 0.942 |
| GAD-7 ≥ 5 | 12/45 (26.67) | 20/63 (31.75) | 0.569 | 26/47 (55.32) | 23/52 (44.23) | 0.270 |
| **Sleep quality** |  |  |  |  |  |  |
| PQSI ≥ 6 (3 months postpartum) | 40/52 (76.92) | 57/73 (78.08) | 0.878 | 46/52 (88.46) | 54/58 (93.10) | 0.398 |
| PQSI ≥ 6 (6 months postpartum) | 37/49 (75.51) | 51/70 (72.86) | 0.746 | 41/47 (87.23) | 45/55 (81.82) | 0.453 |
| PQSI ≥ 6 (12 months postpartum) | 30/45 (66.67) | 45/63 (71.43) | 0.596 | 37/47 (78.72) | 40/52 (76.92) | 0.830 |
| **Social support** |  |  |  |  |  |  |
| MSPSS, Mean ± SD | 64.37±1.59 | 63.57±1.31 | 0.699 | 55.81±1.91 | 58.65±2.24 | 0.345 |
| **Co-parenting relationship at 9 months postpartum** | | | | | | |
| BCRS, Mean± SD |  |  |  |  |  |  |
| Co-parenting agreement | 8.64±0.40 | 8.28±0.35 | 0.512 | 6.98±0.43 | 7.60±0.40 | 0.295 |
| Co-parenting closeness | 7.80±0.47 | 7.32±0.38 | 0.434 | 5.77±0.48 | 6.40±0.45 | 0.336 |
| Co-parenting support | 7.86±0.52 | 6.35±0.42 | **0.025** | 6.07±0.58 | 6.33±0.53 | 0.743 |
| Endorse partner parenting | 9.34±0.41 | 8.63±0.40 | 0.240 | 7.44±0.49 | 8.17±0.44 | 0.269 |
| Division of labor | 7.86±0.48 | 8.12±0.36 | 0.667 | 7.21±0.52 | 7.25±0.41 | 0.950 |
| Exposure to conflict | 2.73±0.39 | 2.09±0.24 | 0.145 | 3.09±0.37 | 2.96±0.29 | 0.778 |
| Co-parenting undermining | 2.41±0.41 | 2.59±0.31 | 0.723 | 3.58±2.88 | 2.79±0.39 | 0.177 |
| **Parenting satisfaction at 9 months postpartum** | | | | | | |
| PSI-SF < 90 | 31/44 (70.45) | 46/68 (67.65) | 0.754 | 19/43 (44.19) | 31/52 (59.62) | 0.134 |
| **Infant development (DDST)** |  |  |  |  |  |  |
| 3 months postpartum |  |  |  |  |  |  |
| Normal | 42/51 (82.35) | 55/73 (75.34) | 0.566 | 40/51 (78.43) | 33/51 (64.71) | 0.119 |
| Suspicious | 8/51 (15.69) | 17/73 (23.29) |  | 9/51 (17.65) | 18/51 (35.29) |  |
| Abnormal | 1/51 (1.96) | 1/73 (1.37) |  | 2/51 (3.92) | 4/51 (7.84) |  |
| 6 months postpartum |  |  |  |  |  |  |
| Normal | 29/48 (60.42) | 40/67 (59.70) | 0.909 | 25 (54.35) | 24 (46.15) | 0.689 |
| Suspicious | 17/48 (35.42) | 23/67 (34.33) |  | 19 (41.30) | 26 (50.00) |  |
| Abnormal | 2/48 (4.17) | 4/67 (5.97) |  | 2 (4.35) | 2 (3.85) |  |
| 12 months postpartum |  |  |  |  |  |  |
| Normal | 37/45 (82.22) | 46/63 (73.01) | 0.079 | 34/45 (75.56) | 34/49 (69.39) | 0.146 |
| Suspicious | 5/45 (11.11) | 16/63 (25.40) |  | 10/45 (22.22) | 9/49 (18.37) |  |
| Abnormal | 3/45 (6.67) | 1/63 (1.59) |  | 1/45 (2.22) | 6/49 (12.24) |  |
| GAD-7, Generalized Anxiety Disorder; PSQI, Pittsburgh Sleep Quality Index; MSPSS, Multidimensional Scale of Perceived Social Support; PSI-SF, Parenting Stress Index-Short Form; BCRS, Brief Co-parenting Relationship Scale; DDST, Denver Development Screen Test; IQR, Inter-quartile range; SD, standard deviations. | | | | | | |
